# Regulation of CYP2J2 and EET Levels in Cardiac Disease and Diabetes

**DOI:** 10.3390/ijms19071916

**Published:** 2018-06-29

**Authors:** Theresa Aliwarga, Eric A. Evangelista, Nona Sotoodehnia, Rozenn N. Lemaitre, Rheem A. Totah

**Affiliations:** 1 Department of Medicinal Chemistry, University of Washington, Seattle, WA 98101, USA; tessa629@uw.edu (T.A.); evangelista.ea@gmail.com (E.A.E.); 2 Cardiovascular Health Research Unit, University of Washington, Seattle, WA 98195, USA; nsotoo@u.washington.edu (N.S.); rozenl@uw.edu (R.N.L.); 3 Department of Medicine, University of Washington, Seattle, WA 98195, USA; 4 Division of Cardiology, University of Washington, Seattle, WA 98195, USA

**Keywords:** CYP2J2, epoxyeicosatrienoic acids, hypertension, cardiovascular disease, diabetes

## Abstract

Cytochrome P450 2J2 (CYP2J2) is a known arachidonic acid (AA) epoxygenase that mediates the formation of four bioactive regioisomers of *cis*-epoxyeicosatrienoic acids (EETs). Although its expression in the liver is low, CYP2J2 is mainly observed in extrahepatic tissues, including the small intestine, pancreas, lung, and heart. Changes in CYP2J2 levels or activity by xenobiotics, disease states, or polymorphisms are proposed to lead to various organ dysfunctions. Several studies have investigated the regulation of CYP2J2 and EET formation in various cell lines and have demonstrated that such regulation is tissue-dependent. In addition, studies linking CYP2J2 polymorphisms to the risk of developing cardiovascular disease (CVD) yielded contradictory results. This review will focus on the mechanisms of regulation of CYP2J2 by inducers, inhibitors, and oxidative stress modeling certain disease states in various cell lines and tissues. The implication of CYP2J2 expression, polymorphisms, activity and, as a result, EET levels in the pathophysiology of diabetes and CVD will also be discussed.

## 1. Introduction

Cytochrome P450 (CYP) is a superfamily of membrane-bound, NADPH-dependent heme-containing monooxygenases involved in the oxidation of both xenobiotics and endogenous compounds. CYPs insert an oxygen atom from molecular oxygen into their product while reducing the second atom to water. CYP2J2 is the only member of the human CYP2J sub-family and one of the major arachidonic acid (AA) epoxygenases. The CYP2J2 gene contains nine exons and eight introns spanning approximately 40.3 kilobases (kb), including about 6 kb of a 5′-flanking region and about 1 kb of 3′-untranslated region [1] (GenBank accession number AF272142). This gene encodes for 502 amino acids, which translate to a protein product approximately 58 kDa. CYP2J2 is primarily expressed in the heart, and to a lesser degree, in the liver, kidney, skeletal muscle, lung, brain, pancreas, and gastrointestinal tract [1,2,3]. Protein expression of CYP2J2 in the human heart is highly variable in contrast to reportedly stable expression in the liver [2]. Similar to many CYP isozymes, CYP2J2 is polymorphically expressed throughout the population. However, the identified polymorphisms are relatively rare and seem to be ethnic-specific, with the exception of CYP2J2*7. This SNP (rs890293) is associated with a G > T substitution in the promoter region (−50 bp), which results in reduced binding of transcription factor Sp1 [1]. CYP2J2*7 was discovered in different ethnic groups with an allelic frequency ranging from 1.1–17% [1,4]. In Caucasian populations, studies show that the CYP2J2*7 allele results in 40% lower protein expression, without significant changes in enzyme activity using ebastine or astemizole as substrates [5]. The presence and different allelic frequencies of CYP2J2*7 among various ethnic groups can, therefore, alter the risk of developing cardiovascular disease (CVD) as summarized in Table 1. The different findings in Table 1 and the reasons for the conflicting results are further discussed in Section 3.1.1.

Several xenobiotics, including ritonavir, astemizole, ebastine, terfenadine, amiodarone, diclofenac, bufurarol, dasatinib, nilotinib, and sorafenib, were identified as CPY2J2 substrates [2,6,7,8,9,10,11,12]. However, apart from ebastine, the contribution of CYP2J2 to drug clearance is not significant, because most substrates are also metabolized by CYP3A4. As a result, most of the research on CYP2J2 is focused on its ability to oxidize AA to four bioactive regioisomers of *cis*-epoxyeicosatrienoic acids (EETs) in vivo [2,13].

**Scheme 1:**
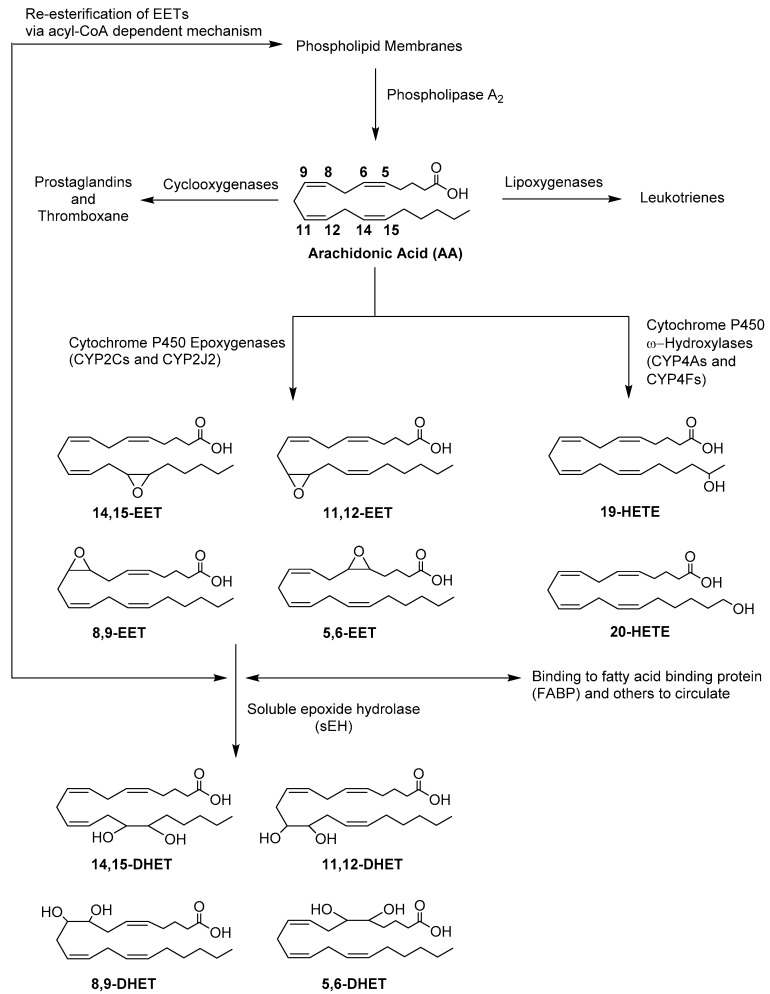
AA metabolic pathway focused on the formation of cytochrome P450 (CYP)-mediated eicosanoids. Upon activation of phospholipase A_2_, arachidonic acid (AA) is released from the phospholipid membrane. AA can be metabolized by cyclooxygenases to generate prostaglandins and thromboxane or by lipoxygenases to form leukotrienes. The CYP ω-hydroxylases, namely CYP4A and CYP4F, sub-families will convert AA to either 19-hydroxyeicosatetraenoic acid (19-HETE) or 20-HETE, while CYP epoxygenases, namely CYP2Cs and CYP2J2, will mediate biotransformation of AA to exclusively four regioisomers of *cis*-epoxyeicosatrienoic acids (EETs). Once formed, EETs can be incorporated back into the phospholipid membrane via acyl-CoA dependent mechanism, bind to other proteins and circulate, or are hydrolyzed by soluble epoxide hydrolase (sEH) to generate dihydroxyeicosatrienoic acids (DHETs).

Arachidonic acid is a 20 carbon ω-6 polyunsaturated fatty acid with four *bis*-allylic *cis*-double bonds. AA is predominantly esterified at the *sn*-2 position in phospholipids with greater amounts in phosphatidylcholine [28]. Concentrations of free AA are variable, ranging between 2.7–50 μM in human plasma [29,30]. Most AA circulates bound to proteins, including albumin, fatty acid binding protein (FABP), and low-density lipoprotein [31,32,33]. Concentrations of esterified AA were determined to be approximately 5 mM per volume (the per volume calculation, used by the authors, was performed by estimating 30 μg of AA in a billion platelets in total volume of 20 μL), 3 pmol/1 million leukocytes, and 15 μM in resting human platelets, leukocytes, and islets of Langerhans, respectively [34,35,36]. Augmented AA esterified in the membrane increases membrane permeability in adult Wistar rat heart mitochondria and increases membrane fluidity in fresh rat aorta myocytes [37,38]. The addition of AA to diet improved cognitive function in healthy, elderly men with low serum AA and synaptic plasticity in aged rats [39,40]. AA has also been reported to modulate ion channels, and an increase in intracellular free AA can trigger apoptosis [41,42]. Lastly, AA is a substrate for cyclooxygenases, lipoxygenases, and CYP metabolic pathways. Through the CYP pathway, AA can be metabolized to 19-hydroxyeicosatetraenoic acid (19-HETE) and 20-HETE by ω−hydroxylases, primarily the CYP4A and CYP4F families. Most pertinent to this review, AA is biotransformed to the bioactive epoxyeicosatrienoic acids (EETs) by CYP epoxygenases, especially CYP2J2 (Scheme 1).

CYP epoxygenases, mainly the CYP2C sub-family and CYP2J2, are reported to form exclusively one or more of the four possible *cis*-EETs. Several other isozymes including CYP3A4, CYP1A1, CYP1A2, CYP2A6, CYP2B6, CYP2D6, CYP2E1, and CYP4X1 [43] have also been reported to catalyze the formation of EETs to some extent. Once formed, EETs can be hydrolyzed to dihydroxyeicosatrienoic acids (DHETs) by soluble epoxide hydrolase (sEH) [44], incorporated back into the phospholipid membrane via acyl-CoA dependent mechanism [45], or bound to FABP to maintain intracellular levels of EETs and prevent hydrolysis by sEH resulting in prolonging duration of action of EETs (Scheme 1) [46]. EETs function both as autocrine and paracrine mediators in the cardiovascular system, kidney, and pancreas. In the cardiovascular system, EETs have been shown to promote angiogenesis, promote hyperpolarization of vascular smooth muscle leading to modulation of vascular tone, and possess anti-inflammatory properties [47,48,49,50].

EETs are putatively believed to bind to and stimulate a receptor(s) activating signaling cascades in order to exert such a range of physiological effects. EETs have been shown to activate various cascades including the MAPK-associated pathways, such as JNK/c-Jun [51] and the PI3K/Akt [52]. There have been several attempts to identify the endogenous EET receptor to date, with a few studies showing EET activation of PPARα [53,54] and therefore control over PPARα regulated genes. Recent work by Park et al. identified the G-protein coupled receptor GPR40, also known as free fatty acid receptor 1 (FFA1), as a possible target for EETs in the vascular system [55]. Specifically, they were able to show that EETs can alter the Ca^2+^ flux in HEK293 cells expressing GPR40 and thus mediate the relaxation of bovine arteries and affect the whole cell potassium currents of HUVEC cells expressing GPR40. Additionally, the effects of EETs in these systems were mitigated by the addition of GPR40 antagonists or calcium-chelating agents [55]. GPR40 is expressed primarily in the pancreas and the brain, at both the mRNA and protein levels [56,57], as well as in the liver, heart, and skeletal muscle at an mRNA level [56,58]. While this finding is exciting and the first report identifying an “EET receptor” more work is needed to determine if the GPR40 is also the receptor for EETs in cardiomyocytes. Most of the work describing GPR40 is focused on its role in the pancreas and the brain. Future efforts should focus on whether EETs bind to GPR40 and exert the EET-mediated cardioprotection, particularly as protein levels of GPR40 are largely unknown in the various sections of the heart.

While the regulatory mechanisms governing many of the hepatic CYP isoforms are well-studied, there is a paucity of information regarding the regulation of *CYP2J2* gene expression. Overexpression of CYP2J2 in experimental animals has been reported to be protective in several disease states, including CVD, whereas CYP2J2 SNPs are associated with risk of incident CVD in humans. In the following sections, we focus on how CVD affects regulation of CYP2J2 in the heart, kidney, and pancreas. We will also present genetic associations of CYP2J2 polymorphisms with related outcomes in humans.

## 2. CYP2J2 Expression and Regulation in the Heart

Several studies reported the robust expression of CYP2J2 in the heart [2,59,60,61]. In non-diseased human hearts, the CYP2J2 protein is observed in the cardiomyocytes and the endothelium of blood vessels via immunohistochemical staining [59]. Although CYP2C9 is highly expressed in endothelial cells compared with CYP2J2, CYP2C9 could only be found in the aorta and coronary arteries of non-diseased human hearts [59,62]. In a model of primary human ventricular myocytes, mRNA expression suggests that CYP2J2 is the dominant CYP epoxygenase in ventricular myocytes, and canonical xenobiotic inducers of CYP enzymes have little to no effect on *CYP2J2* expression [61]. Combined with the primarily extrahepatic expression pattern, this suggests an important endogenous function for CYP2J2.

Few studies have focused on CYP2J2 regulatory mechanisms in cell lines. In HepG2 cells, CYP2J2 is responsive to, and can be upregulated via, the c-Jun/Nrf2 pathway, while treating ventricular myocytes with butylated hydroxyanisole resulted in only a modest and insignificant increase in gene expression [61,63,64].

While typical CYP inducers seem to have little to no regulatory effect, studies have shown that disease states can alter CYP2J2 expression. Bystrom and colleagues demonstrated that *CYP2J2* expression can be induced in human peripheral blood mononuclear cells in response to bacterial lipopolysaccharide [65]. In human cardiomyocytes, our group has shown that expression can be upregulated by reactive oxygen species either directly or by treating with doxorubicin [66]. Studies performed in human first-trimester trophoblast-derived cell lines showed that angiotensin-II and hypoxic condition did not alter CYP2J2 expression; however, tumor necrosis-α (TNF-α), which is elevated in preeclampsia, increased protein expression [67] (Table 2). The involvement of several factors that govern *CYP2J2* expression hint at tissue-dependent variation, but it is important to note that these are in vitro experiments using different cell types and treating with relatively high concentrations of effectors. More robust studies to determine the regulation of this enzyme in healthy and diseased cell lines and tissues will be more informative.

## 3. CYP2J2 and CVD

CVD encompasses a wide range of disease states involving the heart and blood vessels. These include, but are not limited to atherosclerosis, myocardial infarction (MI), cardiac ischemia-reperfusion, heart failure, and arrhythmias, as well as valve problems. These various disease states lead to impaired heart function with serious and often fatal consequences. For the purposes of this review, CVD will be split into two major subcategories: non-ischemic and ischemic cardiomyopathies.

In recent years, interest has steadily increased in the role of CYP2J2 in CVD due to its high expression in the heart, making it the primary route of bioactivation of AA into cardioprotective EETs [2,59,60]. Many studies show a positive role for CYP2J2 in preventing/slowing progression of these conditions in animal models (see below). The role of EETs in protecting against CVD is the primary focus of many of these studies, which highlight the protective effects of elevating EET levels on the outcomes of CVD. It is important to note that while elevation of EET levels were achieved either by the overexpression of CYP2J enzymes or the inhibition/downregulation of sEH, we will focus primarily on studies where the effects of CYP2J2 overexpression on CVD progression were investigated and as summarized in Table 3.

### 3.1. Ischemic Cardiomyopathy

Ischemic cardiomyopathy is defined as CVD resulting from a period of low oxygen flow to the heart. This could be due to a blockage resulting in limited blood flow, and consequently oxygen, to the heart. Reduced oxygen levels lead to a wide range of effects in heart activity and morphology detrimental to proper heart function and homeostasis. Overall, CYP2J2 overexpression in the heart has been shown to improve the outcomes of ischemia and/or ischemia-reperfusion injuries.

#### 3.1.1. Impact of CYP2J2 on Ischemia-Reperfusion Injury and MI

The overexpression of human CYP2J2 in the cardiomyocytes of a C57/BL6 mouse model improves left ventricular recovery post ischemic-reperfusion injury [68]. Cardiac functional recovery was also observed ex vivo in the heart of transgenic mice with cardiomyocyte-specific overexpression of CYP2J2 exposed to 20-min ischemia and 40-min reperfusion, whereas, no recovery was observed in the hearts of transgenic mice with endothelial-specific CYP2J2 [69]. These observations imply that cardiomyocyte CYP2J2, and not endothelial CYP2J2, protects the hearts from ischemic-reperfusion injury. In an ex vivo study of rat hearts, treatment with an sEH inhibitor in normal, hypertensive, and diabetic hearts minimized cardiac damage associated with ischemic-reperfusion injury presumably by increasing or maintaining EET levels [70]. Interestingly, a study by Chaudhary and colleagues [84] showed that this protection is age-dependent. The group found that young mice (2–3 months) carrying the human CYP2J2 transgene had overall improved heart function post-ischemia when compared with wild-type mice. However, this cardioprotective effect due to the overexpression of CYP2J2 significantly declined in old mice (11–13 months). This study emphasized that aging in general reduced left ventricle function, which then reduced the cardioprotective effect of CYP2J2 expression. Conversely, *cis*-EET levels in erythrocyte membrane of C57/BL6 mice increased with age [13]. Perhaps overexpression of CYP2J2 could not compensate for the stress associated with ischemia compounded with the normal cellular dysfunction related with aging.

A study in high-risk cardiovascular patients in Germany did not find significant association between CYP2J2*7 and the risk of developing MI (Table 1) [15]. However, there were two different studies that found that carrying the CYP2J2*7 allele increased the risk of developing premature MI in a Taiwanese population and MI in a predominantly Caucasian population [14,16]. The CYP2J2*7 allele has also been found to be associated with increased risk of MI in a South Indian population [17]. Two different studies in Chinese Han populations found an association between CYP2J2*7 with increased risk of ischemic stroke [18,19]. The odds ratios obtained in these association studies were adjusted for other cardiovascular risk factors, such as age, gender, blood pressure, blood lipid levels, and body mass index. The conflicting observations among various ethnic groups are somewhat expected due to physiological, environmental, and pathological conditions of the study subjects that could manifest differently among specific ethnic groups. Therefore, measuring the circulating EET levels in carriers of the CYP2J2*7 allele compared with controls may be a better assessment of the effect of CYP2J2*7 on circulating EETs and the risk of CVD.

#### 3.1.2. Coronary Artery Disease (CAD)

Atherosclerosis or CAD is a disease caused by accumulation of plaque in the arteries that carry oxygen-rich blood. These vascular plaques harden over time and constrict the flow of oxygen-rich blood to various tissues in the body. In atherosclerosis-prone apolipoprotein E-deficient mice, recombinant adeno-associated virus-mediated CYP2J2 gene overexpression, which is associated with increased EET levels, prevented the development of high-fat diet induced atherosclerosis [71]. Moreover, higher plasma EET levels were observed in CAD patients compared with healthy volunteers, although, obesity within CAD patients led to lower plasma EET levels [85,86]. A follow-up study from the same group showed that total EET levels in both non-obstructive and obstructive CAD patients were generally lower than their control group (no apparent CAD) [87], which is consistent with mouse studies.

Polymorphisms in the *CYP2J2* gene have also been shown to affect CAD risk and incidence in specific populations. The presence of the CYP2J2*7 allele in an African-American population was associated with significantly lower risk of incident CAD, while an increased risk of CAD along with lower plasma EET levels were observed in a Caucasian population [20,21]. In addition, a large number of subjects with this SNP had greater than 50% coronary stenosis, which was a common arbitrary cutoff point to define CAD in this study [88]. Based on seemingly contradictory reports, it is likely that multiple factors, including environmental, are involved and more studies on the effects of *CYP2J2* polymorphism on CAD are needed especially in different ethnic groups with a large number of subjects.

### 3.2. Non-Ischemic Cardiomyopathy

Historically, non-ischemic cardiomyopathy is described as alteration in morphology and function of the heart. Relatively recently, non-ischemic cardiomyopathy was defined as complex disease of the myocardium associated with mechanical or electrical dysfunction that features abnormal ventricular hypertrophy or dilatation, and non-ischemic cardiomyopathy was considered a multifactorial disorder that is often genetic in nature [89]. Following the classifications of non-ischemic cardiomyopathy reported by Maron et al., we will include drug-induced cardiotoxicity, hypertrophy, and arrhythmia in this section.

#### 3.2.1. Drug-Induced Cardiotoxicity

The roles of cytochrome P450s in drug metabolism have long been well-established. Drug metabolizing CYPs typically have higher expression in the intestines, liver, and kidney compared with other organs. Typically, the isoforms primarily involved in drug clearance are isoforms in the CYP1, CYP2, and CYP3 families. CYP3A4 is responsible for a significant portion of drug clearance in most individuals due to its extensive substrate range and abundant hepatic and intestinal expression. In comparison, Lee et al. showed a similar active site volume between CYP2J2 and CYP3A4 [9]. This observation is derived from a homology model of the CYP2J2 active site constructed from the crystal structures of several CYP2 isoforms [9]. While CYP2J2’s relatively low expression in the liver drastically reduces its role in drug metabolism, particularly in orally ingested drugs, it is still subject to the same concerns of drug interactions and drug-induced toxicities. Kaspera et al. previously demonstrated this in a study investigating CYP2J2’s contribution to ritonavir (RTV) metabolism. Using Simcyp^®^ modeling, they predicted CYP2J2 to be responsible for 2–6% of RTV metabolism, with CYPs 3A4 and 2D6 as the primary metabolizers. Among patients carrying the CYP2D6, poor metabolizer phenotype and/or CYP3A4 irreversible inhibition; CYP2J2’s contribution to RTV turnover increases to more than 20% [12]. This highlights the importance of understanding the role of CYP2J2 in the heart, as drug interactions with this enzyme interfering with EET formation can have serious consequences for cardiac health.

One well-studied drug with known cardiotoxicity and interaction with CYP2J2 is the anticancer drug doxorubicin. Doxorubicin is an effective drug to treat human neoplastic and solid tumors; however, it is also known to cause irreversible, cumulative dose-dependent cardiotoxicity, which can ultimately lead to heart failure. Acute exposure to doxorubicin in transgenic mice that overexpress cardiomyocyte-specific CYP2J2 exhibited lower cardiomyocyte apoptosis and less damage to the function of the left ventricle [72]. It is possible that this effect is due to competitive inhibition of the enzyme, preventing AA bioactivation, as demonstrated by Arnold et al. [90], and higher CYP2J2 levels in transgenic mice mitigate doxorubicin toxicity observed in wild-type mice at the same dose. In the same study, the group also noted that doxorubicin is also able to shift the regiospecificity of CYP2J2 towards AA metabolism [90]. Molecular dynamics simulations carried out in a separate study also by Arnold et al. show doxorubicin can prevent the binding of AA by occupying most of CYP2J2’s active sites [91], and the net effect would be an overall reduction in the EET production and thus, signaling.

In a recent study, cardiomyocytes with *CYP2J2* expression silenced by siRNA were more susceptible to doxorubicin toxicity, likely due to elevated reactive oxygen species resulting from drug exposure [66]. These studies demonstrate the protective properties of CYP2J2 in the heart and that drug interactions involving this enzyme will result in negative consequences. The role of CYP2J2 in cardioprotection is putatively due to EET production; however, the overall downstream events surrounding the mechanism(s) by which EETs exert their effect remain to be fully elucidated. It is highly likely that more than one pathway is affected and/or triggered by the release of EETs and studies focused on understanding which pathways are activated when reactive oxygen species rise in the cell are needed.

#### 3.2.2. Hypertrophy and Arrhythmias

Cardiac hypertrophy is a condition characterized by change in the size of cardiomyocytes in response to changes to blood load and blood flow. A few reports have demonstrated that increased CYP2J2 activity and EET production mitigates cardiac hypertrophy [73,75,92]. These situations can be physiological, as in increased exercise over an extended period, or they can be pathophysiological, as a result of hypertension and cardiomyopathy [93]. Wang et al. showed that when hypertension is induced in mice through chronic treatment with angiotensin-II, overexpression of CYP2J2 mitigated cardiac hypertrophic effects of hypertension [73]. They also showed that this protection against hypertrophy was mediated though the AMPKα2 and Akt1 pathways. Mice lacking the AMPKα2 gene were not protected from hypertension-induced hypertrophy. The authors proposed that the protective mechanism was through the reduction of the oxidative stress-mediated NF-κB pathway via PPAR-γ-activation or by activating AMPKα2, which further stimulated the nuclear translocation of Akt1 [73,75,92]. The same group also showed that cardiomyocyte-specific overexpression of CYP2J2 in mice reduced damage associated with cardiac hypertrophy and heart failure and prevented endoplasmic reticulum (ER)-stress and subsequent apoptosis that occurred in heart failure [75].

Another consequence of cardiac hypertrophy is the development of cardiac arrhythmias [94]. The heart’s electrophysiology is governed by various ion channels. While there is an abundance of studies demonstrating the effect of EETs on several ion channels in the heart, fewer studies have demonstrated specifically that CYP2J2 levels can affect cardiac electrophysiology [95,96,97,98,99,100]. One study by Lu et al. found that when CYP2J2 is overexpressed in transgenic mouse hearts, basal K_ATP_ currents were almost two-fold greater when compared with wild-type mice [101]. The group also found that this effect could be attenuated by the addition of CYP epoxygenase inhibitors [101]. Another study demonstrated that overexpression of cardiac CYP2J2 in a transgenic mouse model lowers arrhythmia susceptibility in cardiac hypertrophy [74]. Specifically, CYP2J2 transgenic mice had lessened susceptibility to develop ventricular and atrial arrhythmias following chronic pressure induced hypertrophy and chronic β-adrenergic stimulation, respectively [74]. In addition, mice overexpressing CYP2J2 in cardiac tissue have shorter action potential durations than their wild-type counterparts [97]. This shortened action potential duration was attributed, by Ke and colleagues, to the action of EETs on the maximal peak transient outward K^+^ channel currents, which they reported could be prolonged to match the wild-type counterpart by exposure to MS-PPOH, an epoxygenase inhibitor. Taken together, these studies show that cardiac-specific CYP2J2 expression has beneficial effects on the electrical homeostasis of the ventricles, although a comprehensive mechanistic approach is needed to determine the importance of EETs in maintaining normal electrical function.

## 4. Protective Role of CYP2J2 in the Kidney

A limited number of studies reported on CYP2J2 expression in the kidney. CYP2J2 protein was found in the human kidney at moderate levels, while its transcript was barely detectable [2]. To date, Wu’s study is the sole report on CYP2J2 protein expression in human kidney. An animal study by Chen et al. demonstrated that CYP2J2 expression could be detected in the kidneys of transgenic mice overexpressing CYP2J2 in the endothelium [82]. This suggests that human CYP2J2 may be expressed in human renal endothelium; however, more focused studies are needed to confirm this finding at the mRNA, protein, and activity levels.

Endothelial-specific overexpression of CYP2J2 in streptozotocin-induced diabetic mice attenuated renal damage by minimizing the excretion of albumin and scarring of the glomeruli. In addition, endothelial-specific overexpression of CYP2J2 mitigated the activation of the TGF-β/Smad signaling pathway, which was altered in diabetic mice. In order to confirm the mechanism of diabetic nephropathy observed in the mouse model, this group treated human renal proximal tubular cells with TGF-β1. They found that TGF-β1 inhibited E-cadherin expression while activating the Smad pathway, which further induced renal tubular fibrosis and induced tubular epithelial-mesenchymal transdifferentiation, which was then prevented by the addition of exogenous EETs [82]. In a chronic kidney disease mouse model, increasing EET levels, by chemical inhibition of sEH, led to a decrease in both TGF-β1 and p-Smad3 and induction of PPAR-γ activity [102]. This study and a study by Kawai et al. [103] suggested that EETs acted as a PPAR-γ agonist, which led to a decrease in expression of TGF-β1 and p-Smad3 and therefore, attenuated renal damage.

CYP2J2 gene therapy improved systolic blood pressure by increasing the expression of atrial natriuretic peptide in spontaneously hypertensive rats [76]. In monocrotaline-induced pulmonary hypertensive Sprague–Dawley rats, CYP2J2 gene therapy also attenuated development and vascular remodeling associated with pulmonary hypertension [77]. In a rat model of chronic kidney failure, the overexpression of CYP2J2 via a recombinant adeno-associated viral vector was able to protect further injury to the remaining kidney by inhibiting apoptosis and fibrosis [78]. Another study on CYP2J2 gene delivery via a recombinant adeno-associated virus in mice suppressed adventitial remodeling and inflammation and hypertension induced by angiotensin-II [104]. Overexpression of aortic CYP2J2 via a recombinant adeno-associated virus in angiotensin-II induced abdominal aortic aneurysm apolipoprotein E-deficient mice led to higher EET levels, which activated PPAR and inhibited inflammatory responses [79].

Total plasma EET levels in 10 patients with renovascular disease were significantly lower than 10 normotensive patients [105]. Due to EETs (especially 11,12-EET) functioning as the endothelial-derived hyperpolarizing factor in modulating vascular tone, alteration of EET levels in renovascular disease can be associated with an increase in blood pressure. Therapy using an EET analog or sEH inhibitor might attenuate renal injury and improve blood pressure. A few studies on EET analogs in rat models seemed to ameliorate cisplatin-induced nephrotoxicity and renal injury associated with radiation [106,107]. EET analogs also exhibited a protective effect against renal fibrosis by reducing the endothelial-to-mesenchymal transition in a renal fibrosis mouse model [108]. The administration of sEH inhibitors in angiotensin-II-induced hypertensive rats exhibited improvement in vascular function, blood pressure, and attenuation in renal injury [109,110]. However, a study in a 5/6-nephroectomy mouse model failed to show the protective effect of sEH inhibition [111]. This last study infers that a relatively proper functioning kidney is required to get the protective effect of sEH inhibition.

Carrying a CYP2J2*7 allele was not associated with increased risk of hypertension in African-American subjects [22,23]. An association between CYP2J2*7 and an increased risk of hypertension was found in a Caucasian population in Tennessee, in a Chinese Han population, in a Russian population, and in Saudi Arabian population but not in middle-aged Swedes and South Indian populations (Table 1) [17,23,24,25,26,27]. The CC genotype of another SNP (rs2280275) has been suggested to be a genetic marker for risk of essential hypertension in an Uygur population but not in a Han population [112]. The CYP2J2*7 SNP has also been reported to affect the renal function and the risk of adverse events associated with tacrolimus and mycophenolate sodium in kidney transplant patients in Brazil [113]. Although not conclusive, CYP2J2 genetic variation may reduce EET levels, which could potentially lead to hypertension.

## 5. CYP2J2, EETs, and Risk of Diabetes

Diabetes is characterized by dysfunctions in the body’s ability to produce (type I) or respond to (type II) the hormone insulin [114]. The resulting consequences for the body are that it ultimately becomes hyperglycemic due to chronically high blood glucose levels. As of 2014, over 422 million people live with diabetes worldwide, and as of 2015, 30.3 million people in the United States alone have diabetes [115,116]. In adults, 90% of diabetics have type II diabetes. Diabetes is a chronic disorder associated with long-term complications including CVD, retinopathy, neuropathy, and kidney disease [115,116].

Animal studies have consistently, and overwhelmingly, shown a protective role for EETs, and thus CYP2J2, in the etiology and progression of diabetes [80,81,117]. Ma and colleagues demonstrated that mice carrying the transgene for cardiac-specific overexpression of human CYP2J2 showed improved glucose and insulin plasma levels, as well as improved glucose tolerance and uptake compared to their wild-type counterparts when challenged with a high fat diet and streptozotocin exposure [80]. This study also went on to demonstrate that cardiac CYP2J2 overexpressing mice were protected from the cardiovascular consequences of diabetes, in particular myocardial hypertrophy [80]. They attributed these effects to the activation of the PPAR-γ and MAPK pathways, along with higher atrial natriuretic peptide (ANP) production [80]. This was followed by a study, which demonstrated that CYP2J2 overexpression resulted in attenuated inflammatory responses in isolated hepatocytes and in diabetic mice [81]. Inflammatory pathways leading to elevated cytokine levels are thought to be an important factor in the development of type II diabetes [81,117,118]. In addition, Li et al. also showed that CYP2J2 expression activated the PPAR-γ pathway, which they reasoned could lead to decreased dyslipidemia in their animal models by increasing adipogenesis [81]. Together, these studies demonstrate a clear role for CYP2J2-mediated production of EETs in the prevention of diabetes and its cardiovascular consequences. Efforts to increase production, or decrease the metabolism of EETs are therefore potential therapeutic strategies to treat diabetes.

### CYP2J2 in the Pancreas

The pancreas has a central role in the etiology of diabetes and therefore, the consequent CVD. The pancreas, specifically the β cells in the Islets of Langerhans, is responsible for the metered release of insulin into the bloodstream due to elevated blood glucose levels. As glucose levels rise, increased glycolysis in these cells cause a rise in intracellular ATP levels, the downstream effect of which is that intracellular Ca^2+^ concentrations rise and insulin is released into the bloodstream [114,119]. Diabetes, both type I and type II, is characterized by issues involving the health of the β cells. Type I diabetes is typically characterized by autoimmune destruction of these cells and thus, the reduced ability of the pancreas to produce insulin in response to glucose in the blood. Type II diabetes is putatively due to the increasing resistance of peripheral tissues to respond to the insulin produced by the pancreas but may also be due to the increased resistance of the β cells to high glucose levels in the bloodstream and apoptotic β-cell death [114,120]. Together, these two causes result in reduced insulin release into the bloodstream, as well as decreased response by peripheral tissues to the insulin signal.

Evidence supporting a protective role of CYP2J2 and EETs in the pancreas is limited to a few studies. In 1997, Zeldin et al. reported findings from a study where the authors detected CYP2J2 in human pancreatic tissue, as well as the rat homolog, CYP2J3, in rat pancreatic tissue. Immunoblots using CYP2J specific rabbit anti-human antibodies showed that CYP2J2 expression in the human pancreas is localized to the cells in the Islet of Langerhans [121]. CYP2J2 is proposed to be one of the primary epoxygenases in the human pancreas due to the significant correlation obtained between CYP2J2 protein and extracted total EETs [121]. The effect of CYP2J2 overexpression on β-cell health has not been studied; however, previous reports showed that raising EET levels can reduce Islet β-cell apoptosis. Luo et al. showed that impairment of sEH has positive outcomes in a diabetic mouse model [122]. In addition to reducing β-cell death, sEH knockout or inhibition improved glucose tolerance and insulin secretion [122].

Improvements in blood glucose level, insulin levels, and inflammation markers were observed in diet-induced obese mice that express endothelial-specific CYP2J2 [83]. CYP2J2 gene therapy in diabetic mice significantly improves metabolic function and insulin sensitivity of diabetic mice by altering the expression of enzymes involved in maintaining glucose homeostasis [81]. In a separate study, CYP2J2 gene introduction, along with the administration of an sEH inhibitor, in mice fed with a high fat diet suggests that higher EET levels promote better metabolic function, insulin sensitivity, and reduce inflammation associated with a high fat diet [117]. Further mechanistic studies on how EETs improved metabolic function in mice fed with a high fat diet are needed and will provide insights into therapeutic strategies for obesity-induced metabolic diseases. Perhaps therapy using EET analogs or sEH inhibitors to maintain EET levels will be useful in preventing extensive damage associated with diabetes. Treatment with an sEH inhibitor was shown to prevent diabetic retinopathy in diabetic mice [123]. A dual sEH inhibitor and PPAR-γ agonist are able to attenuate renal injury in metabolic syndrome rat model [124], which is very promising for future therapies involving EETs and maintaining EET levels in the pancreas.

## 6. Conclusions

There is mounting data supporting the protective role CYP2J2 plays in CVD and diabetes. The expression pattern indicates an important function for this specific isozyme in maintaining homeostatic balance through the production of EETs. Most of the evidence for protection stems from the transgenic mouse model with human CYP2J2 overexpression in the cardiomyocytes but not the endothelial cells. Given the evidence to support its role as a cardioprotective enzyme, CYP2J2 presents an attractive target for therapeutic intervention. However, there are obstacles that must be overcome for this to be a viable option. As previously mentioned, CYP2J2 is a drug-metabolizing enzyme that shares a wide range of substrates and inhibitors with other drug-metabolizing CYP isoforms [9,10]. It is important to screen drug candidates for their ability to interact with CYP2J2 and avoid compounds that inhibit CYP2J2 and can cause potential drug-endogenous AA interactions specifically in the heart. Chemical inhibition of cardiac CYP2J2 resulting in lower EET levels could eventually precipitate cardiotoxicity. Increasing expression or activity of CYP2J2 presents another difficulty in targeting this enzyme. Ideally, the goal would be to increase CYP2J2 expression in order to leverage the protective effects of EETs in a cardiac setting. To date, there is no report of a *CYP2J2* inducer suitable as a diagnostic tool to increase CYP2J2 expression in cell or animal models and elucidate the mechanism(s) through which it exerts its protective effect under various stress conditions. The data available suggests tissue dependence, as well as tight regulation with resistance to pharmacological up-regulation. A few studies using recombinant adeno-associated viruses to increase the expression of CYP2J2, which in turn improve cardiac or renal damage, have been successful in various murine models [51,78,79]. This therapeutic avenue is therefore attractive but may not be attainable to increase CYP2J2 expression in humans. In contrast with the abundance of data from animal and in vitro studies, human studies on EETs and disease outcomes are scarce and largely limited to genetic associations. Human studies using large data samples investigating the association between EETs and the risks of incident diabetes and CVD are needed.

## Figures and Tables

**Table 1:** Summary of the effect of CYP2J2*7 in altering the risk associated with developing cardiovascular dysfunction in different ethnic groups.

Disease State	Population	Risk	Significant Association	References
Premature myocardial infarction	Taiwanese	Increased	Yes	[14]
Myocardial infarction (MI)	Germanic	None	/	[15]
	Caucasian in western Washington state	Increased	Yes	[16]
	South Indian	Increased	Yes	[17]
Ischemic stroke	Chinese Han	Increased	Yes	[18,19]
Atherosclerosis	African-American	Decreased	Yes	[20]
	Caucasian from central Germany	Increased	Yes	[21]
Hypertension	African-American	None	/	[22,23]
	Caucasian in Tennessee	Increased	Yes	[23]
	Chinese Han	Increased	Yes	[24]
	Russian	Increased	Yes	[25]
	Saudi Arabian	Increased	Yes	[26]
	Middle-aged Swedes	None	/	[27]
	South Indian	None	/	[17]

**Table 2:** Summary of CYP2J2 expression by some effectors in various cell lines.

Source	Type of Effectors	Effect on CYP2J2 Expression	References
Primary human ventricular myocytes	Chemical	Little *	[61]
	Reactive oxygen species	Increased *	[66]
HepG2 cells	Chemical	Increased	[63,64]
Peripheral human mononuclear cells	Bacterial lipopolysaccharides	Increased	[65]
Human, first-trimester trophoblast-derived cells	Angiotensin-II	None	[67]
	Hypoxia	None	[67]
	TNF-α	Increased	[67]

* indicates mRNA expression. TNF-α: tumor necrosis-α.

**Table 3:** The effects of CYP2J2 overexpression on diabetes and cardiovascular disease (CVD) progression and recovery.

Disease State	Model	Condition or Treatment	Effects	References
Ischemic-reperfusion injury	Mouse overexpressing cardiac-specific CYP2J2	Isolated perfused heart	Improved left ventricular recovery	[68]
	Mouse overexpressing endothelial-specific CYP2J2	Isolated perfused heart	No improvement on left ventricular function	[69]
Ischemic-reperfusion injury and hypertension	Wistar rat	sEH inhibitor	Minimized cardiac damage	[70]
Ischemic-reperfusion injury and diabetes	Wistar rat	sEH inhibitor	Minimized cardiac damage	[70]
Atherosclerosis	Apolipoprotein-E deficient mouse	High-fat diet and recombinant adeno-associated virus mediated CYP2J2 expression	Reduced vascular apoptosis	[71]
Doxorubicin-induced cardiotoxicity	Mouse overexpressing cardiac-specific CYP2J2	Acute and chronic doxorubin	Lower cardiomyocyte apoptosis and less damage to left ventricular function	[72]
Hypertrophy	AMPKα2 knockout mouse	Angiotensin-II to induce hypertension, and recombinant adeno-associated virus mediated CYP2J2 expression	Mitigated cardiac hypertrophic effect of hypertension	[73]
	Mouse overexpressing cardiac-specific CYP2J2	Chronic pressure induced hypertrophy via transverse aortic constriction surgery	Reduced ventricular arrhythmia	[74]
	Mouse overexpressing cardiac-specific CYP2J2	Chronic β-adrenergic stimulation by infusion of isoproterenol	Reduced atrial arrhythmia	[74]
Hypertrophy and heart failure	Mouse overexpressing cardiac-specific CYP2J2	Infusion of isoproterenol or angiotension-II	Reduced damage associated with hypertrophy and heart failure	[75]
Hypertension	Spontaneously hypertensive mouse	Recombinant adeno-associated virus mediated CYP2J2 expression	Improved systolic blood pressure	[76]
Pulmonary arterial hypertension	Sprague-Dawley rat	Monocrotaline to induce pulmonary arterial hypertension and CYP2J2 gene delivery	Attenuated development and vascular remodeling	[77]
Chronic kidney failure	5/6 nephrectomized rat	Recombinant adeno-associated virus mediated CYP2J2 expression	Protected remaining renal function	[78]
Abdominal aortic aneurysm	Apolipoprotein-E deficient mouse	Angiotensin-II to induce abdominal aortic aneurysm and recombinant adeno-associated virus mediated aortic-specific CYP2J2 expression	Activated PPAR and inhibited inflammatory responses	[79]
Diabetes	Mouse overexpressing cardiac-specific CYP2J2	Streptozotocin to induce diabetes and high-fat diet	Improved blood glucose and insulin levels, glucose tolerance and uptake, and protected against myocardial hypertrophy	[80]
	Diabetic mouse	Recombinant adeno-associated virus mediated CYP2J2 expression	Improved metabolic function and attenuated inflammatory responses	[81]
Diabetic nephropathy	Mouse overexpressing endothelial-specific CYP2J2	Streptozotocin to induce diabetes	Attenuated renal damage	[82]
Obesity	Mouse overexpressing endothelial-specific CYP2J2	High-fat diet to induce obesity	Improved blood glucose and insulin levels and inflammation markers	[83]
